# Morphology, taxonomy and mating-type loci in natural populations of *Volvox carteri* in Taiwan

**DOI:** 10.1186/s40529-018-0227-9

**Published:** 2018-04-03

**Authors:** Hisayoshi Nozaki, Noriko Ueki, Mari Takusagawa, Shota Yamashita, Osami Misumi, Ryo Matsuzaki, Masanobu Kawachi, Yin-Ru Chiang, Jiunn-Tzong Wu

**Affiliations:** 1 Department of Biological Sciences, Graduate School of Science, University of Tokyo, Hongo, Bunkyo-ku, Tokyo, 113-0033 Japan; 2 Department of Biology, Brooklyn College, City University of New York, Brooklyn, NY 11210 USA; 3 Department of Botany, Graduate School of Science, Kyoto University, Sakyo-ku, Kyoto, 606-8502 Japan; 4 Department of Biological Science and Chemistry, Faculty of Science, Graduate School of Medicine, Yamaguchi University, Yoshida, Yamaguchi, 753-8512 Japan; 5 Center for Environmental Biology and Ecosystem Studies, National Institute for Environmental Studies, Onogawa, Tsukuba, Ibaraki 305-8506 Japan; 6 Biodiversity Research Center, Academia Sinica, Nankang, Taipei, 11529 Taiwan

**Keywords:** Mating-type locus, Morphology, Sexual reproduction, Taxonomy, *Volvox*, *Volvox carteri*, *Volvox carteri* f. *nagariensis*

## Abstract

**Background:**

*Volvox carteri* f. *nagariensis* is a model taxon that has been studied extensively at the cellular and molecular level. The most distinctive morphological attribute of *V. carteri* f. *nagariensis* within *V. carteri* is the production of sexual male spheroids with only a 1:1 ratio of somatic cells to sperm packets or androgonidia (sperm packet initials). However, the morphology of male spheroids of *V. carteri* f. *nagariensis* has been examined only in Japanese strains. In addition, *V. carteri* f. *nagariensis* has heterothallic sexuality; male and female sexes are determined by the sex-determining chromosomal region or mating-type locus composed of a > 1 Mbp linear chromosome. Fifteen sex-specific genes and many sex-based divergent shared genes (gametologs) are present within this region. Thus far, such genes have not been identified in natural populations of this species.

**Results:**

During a recent fieldwork in Taiwan, we encountered natural populations of *V. carteri* that had not previously been recorded from Taiwan. In total, 33 strains of this species were established from water samples collected in Northern Taiwan. Based on sequences of the internal transcribed spacer 2 region of nuclear ribosomal DNA and the presence of asexual spheroids with up to 16 gonidia, the species was clearly identified as *V. carteri* f. *nagariensis*. However, the sexual male spheroids of the Taiwanese strains generally exhibited a 1:1 to > 50:1 ratio of somatic cells to androgonidia. We also investigated the presence or absence of several sex-specific genes and the sex-based divergent genes *MAT3m*, *MAT3f* and *LEU1Sm*. We did not identify recombination or deletion of such genes between the male and female mating-type locus haplotypes in 32 of the 33 strains. In one putative female strain, the female-specific gene *HMG1f* was not amplified by genomic polymerase chain reaction. When sexually induced, apparently normal female sexual spheroids developed in this strain.

**Conclusions:**

Male spheroids are actually variable within *V. carteri* f. *nagariensis.* Therefore, the minimum ratio of somatic cells to androgonidia in male spheroids and the maximum number of gonidia in asexual spheroids may be diagnostic for *V. carteri* f. *nagariensis*. *HMG1f* may not be directly related to the formation of female spheroids in this taxon.

**Electronic supplementary material:**

The online version of this article (10.1186/s40529-018-0227-9) contains supplementary material, which is available to authorized users.

## Background

The genus *Volvox* represents the most complex member of volvocine green algae, especially in terms of multicellularity and sex (Kirk 1998; Hiraide et al. 2013). In recent years, *Volvox carteri* f. *nagariensis* has been studied extensively at the cellular and molecular level (e.g., Kirk et al. 1999; Ferris et al. 2010). Although this taxon or form was originally described based on a natural sample collected in Nagari, India (Iyengar 1933), most of the studied strains are Japanese strains of *V. carteri* f. *nagariensis* such as Eve (Starr 1969; Kirk et al. 1999; Ferris et al. 2010; Geng et al. 2014). Six forms are recognized within *V. carteri*, and the most distinctive morphological attribute for distinguishing *V. carteri* f. *nagariensis* from other forms within this species is the production of sexual male spheroids with only a 1:1 ratio of somatic cells to sperm packets or androgonidia (sperm packet initials) (Nozaki 1988). However, this ratio has been examined only in Japanese strains (Starr 1969; Nozaki 1988; Geng et al. 2014) irrespective of the presence of Indian strains from Poona (Adams et al. 1990).

*Volvox carteri* f. *nagariensis* is an oogamous species that has heterothallic sexuality determined by the sex-determining chromosomal region or mating-type locus where the presence of sex-specific genes (genes harboring in only one of the two sexes) and sex-based divergent shared genes (gametologs; two homologous genes harboring in this regions of both of the two sexes) was recently demonstrated (Ferris et al. 2010). Sex in *V. carteri* f. *nagariensis* is determined based on the presence or absence of such genes. However, sex identification in natural populations of this species has not previously been conducted by using molecular markers. Furthermore, the mating-type locus of *V. carteri* f. *nagariensis* is composed of a > 1 Mbp linear chromosome where recombination is suggested to be repressed (Ferris et al. 2010). Although the mating-type locus or sex chromosomal region does not exhibit recombination in *Chlamydomonas reinhardtii* under the laboratory conditions, recombination and gene conversion of *C. reinhardtii* mating-type locus genes were demonstrated in natural populations of this species (De Hoff et al. 2013). However, recombination of the mating-type locus genes has not been examined in natural populations of *V. carteri*.

During a recent field survey of the freshwater green algae in Taiwan, we encountered natural populations of *V. carteri*, which have not previously been recorded in Taiwan (Yamagishi 1992). Based on the internal transcribed spacer 2 region of nuclear ribosomal DNA (nuclear rDNA ITS-2), the populations were clearly identified as *V. carteri* f. *nagariensis*. This study was undertaken to reveal morphological details of *V. carteri* f. *nagariensis* originating from Taiwan and to examine presence or absence of several sex-specific and sex-based divergent genes in these natural populations of *V. carteri* f. *nagariensis.*

## Methods

Table 1 shows the origins of the 33 Taiwanese strains used in the present study. Clones were established by the pipette-washing method (Pringsheim 1946). The cultures were maintained in screw-cap tubes (18  ×  150 mm) containing 11 mL AF-6 or VTAC medium (Kawachi et al. 2013) at 25 °C with a 14:10 h light:dark (LD) schedule under cool-white fluorescent lamps at an intensity of 80–130 μmol m^−2^ s^−1^. Several of these new wild strains are available from the Microbial Culture Collection at the National Institute for Environmental Studies (Kawachi et al. 2013; http://mcc.nies.go.jp/index_en.html) as NIES-4205–4210 (Table 1). To observe the morphology of asexual spheroids, the cultures were grown in 11 mL VTAC medium in Petri dishes (10 × 60 mm) at 25 °C with a 14:10 h LD cycle. For comparison, we also used Eve [i.e., UTEX 1885, obtained from the Culture Collection of Algae at the University of Texas at Austin (UTEX, https://utex.org)], a typical female strain of *V. carteri* f. *nagariensis* (Ferris et al. 2010; Geng et al. 2014).

**Table 1:** List of Taiwanese strains of *Volvox carteri* f. *nagariensis*

Habitat/date [locality designation]	Longitude, latitude [temperature/pH]	Number of strains	Sex based on genomic PCR	Strains deposited in NIES-collection and their DDBJ/EMBL/GenBank accession numbers of nuclear rDNA ITS-2
Rice paddy/25 May 2016 [2]	N 25°13′02.76′′, E 121°36′22.44′′ [26.1 °C, pH 6.7]	0	Male	
		1	Female	2016-tw-nuk-Chg (= NIES-4205), LC376031
Fallow rice paddy/9 Jun 2016 [4]	N 25°13′51.27′′, E 121°37′53.78′′ [36.2 °C, pH 6.8]	4	Male	
		12	Female	2016-0609-v-1 (= NIES-4206), LC376032
Fallow rice paddy/10 Jun 2016 [6]	N 25°00′52.51′′, E 121°55′33.38′′ [29.7 °C, pH 6.7]	14	Male	2016-0610-v-11 (= NIES-4207), LC376033; 2016-tw-nuk-6-1 (= NIES-4208), LC376034
		0	Female	
Rice paddy/10 Jun 2016 [8]	N 25°01′09.82′′, E 121°55′21.76′′ [29.0 °C, pH 5.7]	1	Male	2016-tw-nuk-8-1 (= NIES-4209), LC376035
		1	Female	2016-tw-nuk-8-2 (= NIES-4210), LC376036

Morphological details of asexual and sexual spheroids were observed mainly in three strains, 2016-0609-v-1 (female), 2016-tw-nuk-6-1 (male) and 2016-tw-nuk-8-1 (male). To observe asexual spheroids and reproduction, we examined small aliquots of asexual spheroids, in actively grown 2- to 5-day-old cultures in the Petri dishes (10 × 60 mm). Sexual male spheroids developed spontaneously in a culture of male strains grown in VTAC medium at 20 °C. To induce sexual spheroids, 0.1–0.2 mL of the sexual inducer (supernatant of the male culture after the formation of sperm packets) and ca. 0.5 mL of an actively growing female or male strain were added to 11 mL of fresh VTAC medium in a tube and placed at 25 °C with a 14:10 h LD cycle. After 2–4 days, sexual spheroids developed. To observe zygotes, sexual male and female spheroids induced in tubes (each 11 mL) were mixed with fresh VTAC medium (11 mL) in Petri dishes (20 × 150 mm) that were placed at 25 °C with a 14:10 h LD cycle.

Light microscopy was carried out using a BX60 microscope (Olympus, Tokyo, Japan) equipped with Nomarski optics. The cells in spheroids were counted as previously described (Nozaki 1988). Individual cellular sheaths of the gelatinous matrix of the spheroids were examined after mixing approximately 10 µL cultured material with 2–5 µL of 0.002% (w/v in distilled water) methylene blue (1B-429 WALDECK GmbH & Co Division Chroma, Münster, Germany).

Extraction of total DNA and sequencing of nuclear rDNA ITS-2 were performed as previously described (Nozaki et al. 2000, 2006; Hayama et al. 2010). Nuclear rDNA ITS-2 sequences of the Taiwanese strains (Table 1) and a Chinese strain “Wang (Shenzhen)” (DDBJ/EMBL/GenBank Accession Number HG422808) and the sequences aligned with the data set in Kawafune et al. (2015) were used for phylogenetic analyses. The alignment of the whole data matrix was refined as described by Kawafune et al. (2015). Since the ITS-2 sequences from all of the 33 Taiwanese strains were identical to those of strains Eve and NIES-397 of *V. carteri* f. *nagariensis*, these 35 strains were treated as a single operational taxonomic unit (OTU). The alignment of 10 OTUs of *V. carteri* and *V. obversus* (Additional file 1: Figure S1) was subjected to the maximum-likelihood (ML) analysis, based on the selected model (T92 + G) with 1000 bootstrap replicates (Felsenstein 1985) using MEGA7 (Kumar et al. 2016). In addition, 1000 bootstrap replicates were performed using the maximum-parsimony (MP) method, based on a branch-and-bound search by PAUP 4.0b10 (Swofford 2002). *V. obversus* was treated as the outgroup because it is sister to *V. carteri* (Kawafune et al. 2015; Nozaki et al. 2015).

We performed genomic polymerase chain reaction (PCR) using the primers listed in Table 2 to check the presence or absence of several sex-specific genes and the sex-based divergent genes *MAT3m*, *MAT3f* and *LEU1Sm* (Ferris et al. 2010) in the 33 Taiwanese strains and Eve. PCR was carried out using KOD FX Neo DNA polymerase (TOYOBO, Osaka, Japan) according to the manufacturer’s instructions.

**Table 2:** Primer sequences and product sizes of sex-specific genes, sex-based divergent genes *MAT3* and *LEU1S* (Fig. 4b), and *ACTIN* gene

Gene name [Accession number]	Primer name	Primer sequence (5′ to 3′)	Product size (bp)
*MID1m* [GU784916]	MID1-F1	GCTGAAGGAGTGTATCGACGCATT	287
	MID1-R1^a^	GCTGCCTGCAAATTCGCTTAAGGT	
*HMG1f* [GU784915]	HMG1-1F	GACTGCTCACCTATCAGTTGGTCA	326
	HMG1-1R^a^	TTGCCACCTCGTATGCATCGAAGT	
*MAT3f* [GU784915]	MAT3f-F	AATCGCATGCAGCGCGCACTTTCT	404
	MAT3f-R^a^	TCAGCTGCCAGGCTGGCAGTTATG	
*MAT3m* [GU784916]	MAT3 m-F	GTGCGCATGCAACGTGCGCTTGAC	401
	MAT3 m-R^a^	TCAGCTGCAAGGTTAGCCGTCCTC	
*FSI1f* [GU784915]	FSI-1-F	ATGCAGCGGATTGAGATGGCCTTC	428
	FSI-1-R^a^	ATCACCTCTGTACAGGTCGCCTCC	
*MTM0946* [GU784916]	MTM-946-F	GCCAACCAACCCGTTCCCAGCATA	420
	MTM-946-R^a^	GCCCTGGACGACTCTAGGGGTCTT	
*MTF0821* [GU784915]	MTF-0821-F	GTCGGGCTCATCAGTAAAAGCTGG	358
	MTF-0821-R^a^	GTTCGCCAAGGAACCGAGCTTAAC	
*LEU1Sm* [GU784916]	LEU1Sm-F	ATGCAGTTTCAGGTCCTAAGGGCT	451
	LEU1Sm-R^a^	CACGTACAGAGTTTCCAAGCCGCT	
*MTF0991* [GU784915]	MTF0991-1F	ATGAACACATGCCCATCCTGGTGC	396
	MTF0991-1R^a^	TCAAGGCGCTGCATCGCTGCTACT	
*MTF2030* [GU784915]	MTF2030-1F	ATGGCAAACGCAGACCCTGGTACA	464
	MTF2030-2R^a^	CCCTTGCGCCTGCCAGCAGACCCA	
*ACTIN* [XM_002955490]	VxCnAct-F2	GAAGCTCTGCTATGTGGCGCTGGA	410^b^
	VxCnAct-R3^a^	ATGGTCGTTCCACCAGAGAGCACG	

^a^ Reverse primer

^b^ Determined in the present study

## Results

### Morphology

**Fig. 1:**
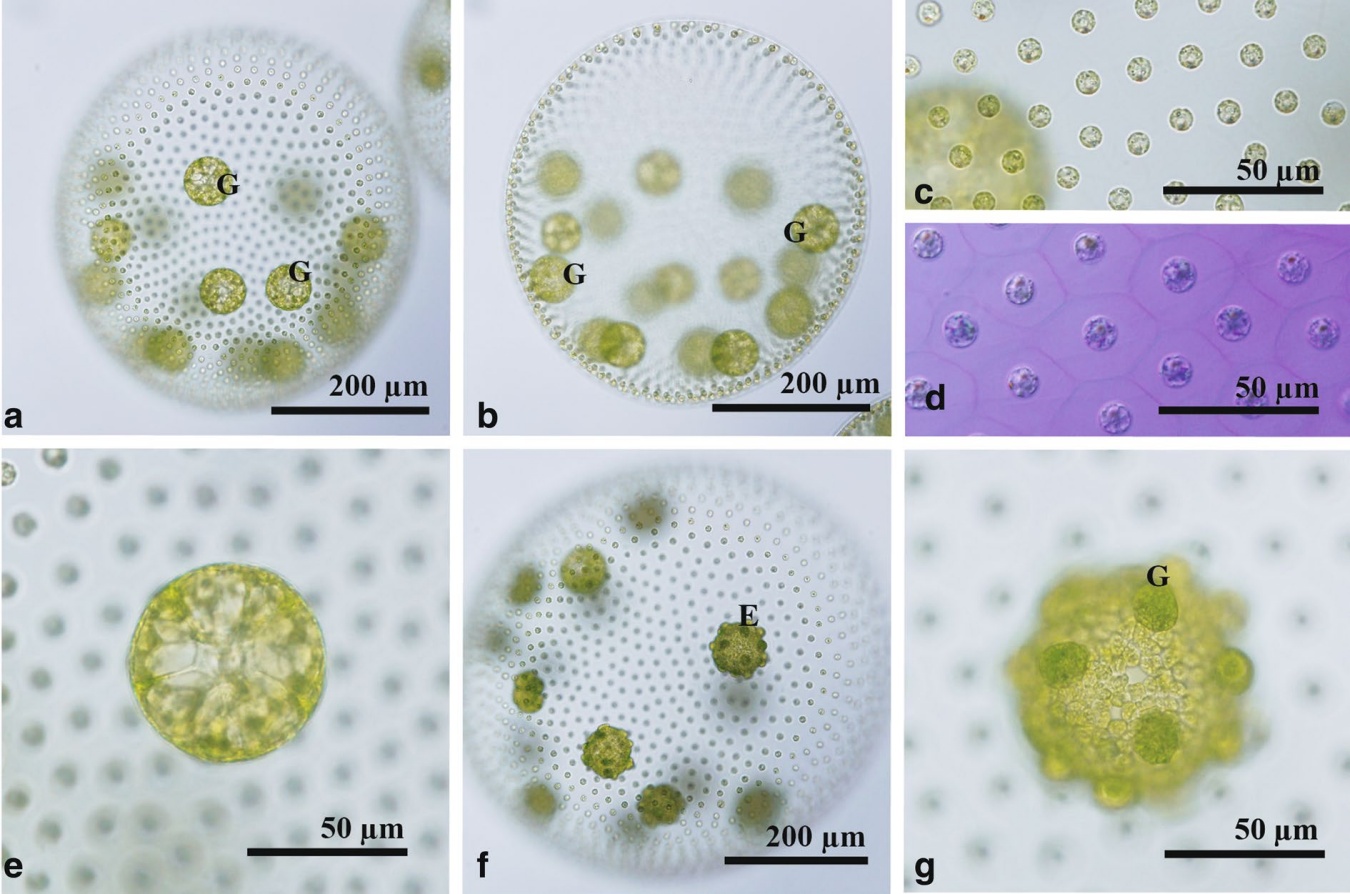
Light microscopy of asexual spheroids in Taiwanese strains of *Volvox carteri* f. *nagariensis*. **a** Surface view of a spheroid showing undivided gonidia (G). 2016-tw-nuk-6-1. **b** Optical section of a spheroid in (**a**) with gonidia (G). **c** Surface view of spheroid. Note no cytoplasmic bridges between somatic cells. 2016-tw-nuk-6-1. **d** Surface view of spheroid showing individual sheaths of the gelatinous matrix. Stained with methylene blue. 2016-tw-nuk-8-1. **e** Optical section of gonidium. 2016-tw-nuk-6-1. **f**, **g** Pre-inversion plakea or embryo (E) showing gonidia (G) of the next generation outside. 2016-tw-nuk-8-1

Asexual spheroids of Taiwanese strains were ovoid or spherical in shape and measured up to 920 μm long; each spheroid contained 1400–3000 somatic cells embedded within the periphery of the gelatinous matrix and large reproductive cells or gonidia in the posterior two-thirds (Fig. 1a, b). Each somatic cell was nearly spherical or ovoid in shape, up to 10 μm in diameter, and enclosed by a rectangular to hexagonal space formed by individual sheaths of gelatinous matrix (Fig. 1c, d). The cells lacked adjoining cytoplasmic bridges (Fig. 1c), and exhibited cup-shaped chloroplasts with a single stigma and a basal pyrenoid. The spheroid exhibited a gradual decrease in stigma size from the anterior to posterior pole. Gonidia were spherical in shape and had large vacuoles, measuring up to 92 μm in diameter (Fig. 1e). There were up to 16 gonidia per spheroid (Fig. 1a, b).

Asexual reproduction occurred as previously described (Starr 1969; Nozaki 1988). Successive divisions of each gonidium resulted in formation of a plakea. Gonidia of the next generation were evident outside the plakea because of the unequal cytokinesis (Fig. 1f, g). The plakea then inverted to form a compact, spheroidal daughter spheroid.

**Fig. 2:**
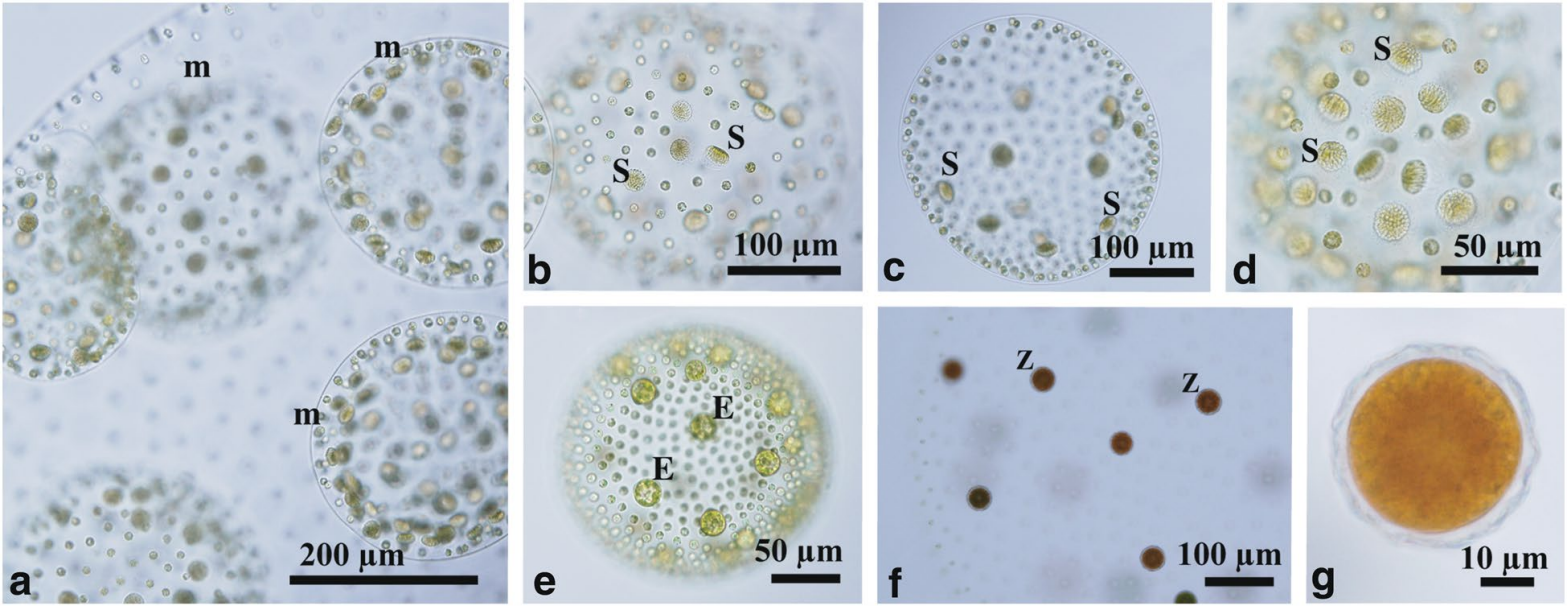
Light microscopy of sexual reproduction in Taiwanese strains of *Volvox carteri* f. *nagariensis*. 2016-tw-nuk-6-1 (male) and 2016-0609-v-1 (female). **a** Male spheroids (m) developing within parental spheroid. **b**–**d** Various ratios of somatic cells to sperm packets (S) in male spheroids. Note 4–12:1 ratio in (**a** and **b**), > 50:1 ratio in (**c**), and 1:1 ratio in (**d**). **e** Female spheroids with eggs (E). **f** Mature zygotes (Z) in female spheroid. **g** Optical section of mature zygote with reticulate wall

Sexual male spheroids were ellipsoidal or ovoid and 128- or 256-celled, containing biflagellate somatic cells and androgonidia that developed into sperm packets (Fig. 2a–d). The number of androgonidia or sperm packets in male spheroids varied even within the same culture. The ratio of somatic cells to androgonidia (sperm packets) in male spheroids was generally 4–12:1 (Fig. 2a, b). However, male spheroids sometimes exhibited a > 50:1 ratio (Fig. 2c) or approximately 1:1 ratio (Fig. 2d) of somatic cells to androgonidia. Female spheroids were ellipsoidal or ovoid in shape, and contained 1000–2000 biflagellate somatic cells and 22–28 eggs (Fig. 2e). The zygotes developed within the female spheroid after possible fertilization (Fig. 2f). The mature zygotes had a reticulate cell wall and were reddish brown in color, measuring 34–40 μm in diameter (Fig. 2g).

Morphology of asexual and sexual spheroids in the present Taiwanese strains was essentially consistent with that of the Japanese strains of *V. carteri* f. *nagariensis* except for male spheroids (Starr 1969; Nozaki 1988). The Japanese strains produce male spheroids with only a 1:1 ratio of somatic cells to androgonidia (Starr 1969; Nozaki 1988). In contrast, male spheroids of the present Taiwanese strains exhibited variability in the ratios (Fig. 2).

### Molecular phylogeny of nuclear rDNA ITS-2

**Fig. 3:**
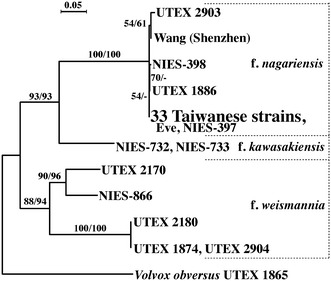
Phylogenetic positions of 33 Taiwanese strains within three forms of *Volvox carteri*. The tree was based on the maximum likelihood (ML) analysis of the data matrix of Kawafune et al. (2015) plus a Chinese strain “Wang (Shenzhen)” (Additional file 1: Figure S1). Numbers at left and right above the branches represent bootstrap values (50% or more) of ML and maximum parsimony analyses, respectively, based on 1000 replicates. The branch lengths are proportional to the evolutionary distances used in the ML analysis and indicated by bar above the tree. For details, see “Methods”

All 33 strains from Taiwan had the same nuclear rDNA ITS-2 sequences, which were also identical to the *V*. *carteri* f. *nagariensi*s strain Eve (UTEX 1885). The phylogenetic analysis clearly demonstrated that the Taiwanese strains belonged to the *V*. *carteri* f. *nagariensi*s clade (Fig. 3).

### Genomic PCR of 33 strains from Taiwan

We screened the 33 Taiwanese strains for the presence or absence of five female-specific genes, two male-specific genes, the female and male types of the sex-based divergent gene *MAT3*, and the male type of *LEU1S* (Ferris et al. 2010) (Tables 1, 2).

**Fig. 4:**
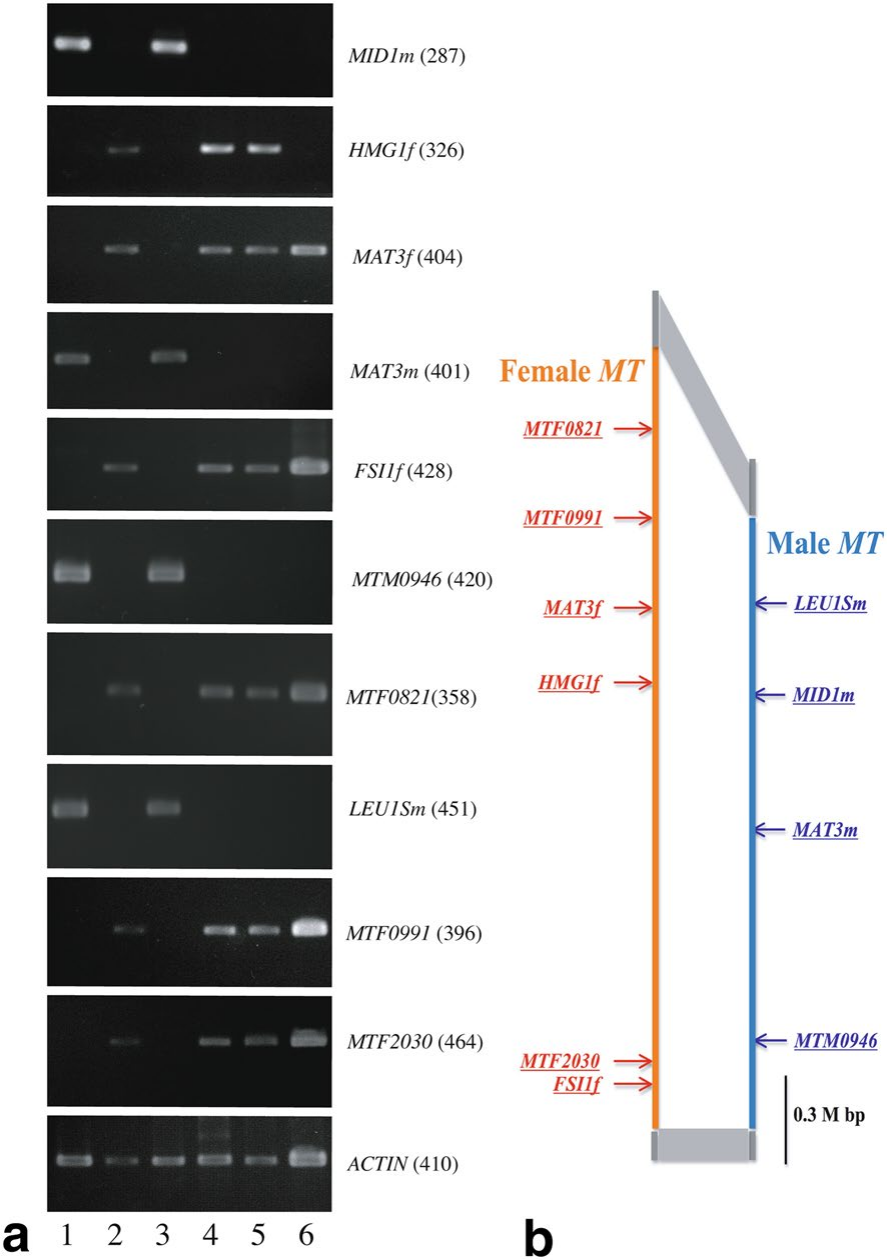
Mating-type locus (*MT*) genes of *Volvox carteri* f. *nagariensis*. **a** Results of genomic PCR of five Taiwanese strains (lanes 1–4 and 6) and Eve (UTEX 1885) (lane 5) using primers listed in Table 2. Numbers within the parentheses just right to the gene designations represent expected lengths of the PCR products. Lane 1: 2016-tw-nuk-6-1. Lane 2: 2016-0609-v-1. Lane 3: 2016-0610-v-11. Lane 4: 2016-tw-nuk-Chg. Lane 6: 2016-tw-nuk-8-2. Note that both male genes and *HMG1f* were not amplified in 2016-tw-nuk-8-2. **b** Diagram showing positions of sex-specific and sex-based divergent genes in the mating-type locus analyzed by genomic PCR in (**a**). Based on Ferris et al. (2010)

Thirty-two of the Taiwanese strains and Eve could be clearly subdivided into female and male types (Table 1). The female type was composed of 13 Taiwanese strains and Eve, which shared the presence of five female-specific genes and *MAT3f* and the absence of two male-specific genes and male types of two sex-based divergent genes (*MAT3m* and *LEU1Sm*) (Table 1, Fig. 4; Ferris et al. 2010). Nineteen other strains belonged to the male type, based on the presence of two male-specific genes, *MAT3m* and *LEU1Sm* and the absence of five female-specific genes and *MAT3f* (Table 1, Fig. 4; Ferris et al. 2010). Thus, there seemed to be no recombination or deletion of such genes between male and female mating-type locus haplotypes in 32 of the 33 Taiwanese strains. However, in the remaining strain (2016-tw-nuk-8-2), one female-specific gene (*HMG1f*) was not amplified whereas this strain contained four other female-specific genes and *MAT3f* and showed no amplifications of all four of the male genes examined (Fig. 4). Thus, this strain seemed to be a female strain without amplification of *HMG1f * in genomic PCR.

### Asexual spheroids and sexual induction of the HMG1f-lacking putative female strain

**Fig. 5:**
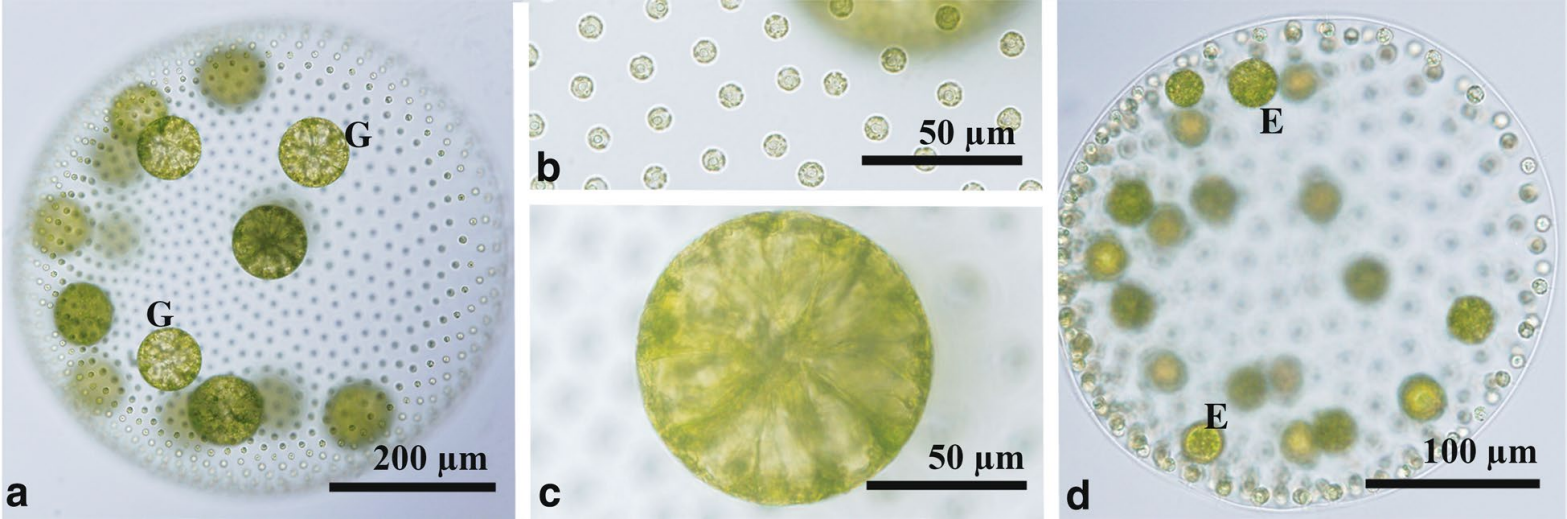
Light microscopy of a putative female strain (2016-tw-nuk-8-2) with no amplification of *HMG1f* in genomic PCR (see Fig. 3 and Additional file 1: Figure S2). **a** Asexual spheroid with 16 gonidia (G). **b** Surface view of asexual spheroid showing no cytoplasmic bridges between cells. **c** Optical section of gonidium in asexual spheroid. **d** Female spheroid with eggs (E)

Asexual spheroids of the *HMG1*-lacking strain (2016-tw-nuk-8-2) were essentially the same as those of other Taiwanese strains, with up to 16 gonidia that developed into daughter spheroids via successive divisions and inversion (Fig. 5a–c). When sexually induced, apparently normal female sexual spheroids developed in this strain. The number of eggs produced was generally 20 or more (Fig. 5d). No morphological differences were recognized between these female spheroids and the female spheroids of other female stains. Attempts to cross 2016-tw-nuk-8-2 with male Taiwanese strains were unsuccessful, because the male Taiwanese strains suddenly lost their ability to form male spheroids during the study (from April to June 2017).

## Discussion

Nozaki (1988) recognized six forms within *V*. *carteri*, and characterized *V. carteri* f. *nagariensis* as having male spheroids with only a 1:1 ratio of somatic cells to androgonidia (sperm packets). However, this ratio was based on observations of only Japanese strains studied by Starr (1969), and subsequent studies have also used only these strains and/or their progenies (e.g., Kirk et al. 1999; Ferris et al. 2010). Based on the ITS-2 phylogeny, the present Taiwanese strains are clearly identified as *V*. *carteri* f. *nagariensis* because these have the exactly the same ITS-2 sequence as that of the Japanese strains (Fig. 3). However, the male spheroids of the Taiwanese strains exhibited a 1:1 to > 50:1 ratio of somatic cells to androgonidia (Fig. 2). Thus, the morphology of male spheroids is actually variable within *V*. *carteri* f. *nagariensis.* On the other hand, *V*. *carteri* f. *nagariensis* is similar to *V*. *carteri* f. *kawasakiensis* with respect to the production of 16 or more gonidia in asexual spheroids. However, the minimum ratio of somatic cells to androgonidia differs between these two forms. The minimum ratio in *V*. *carteri* f. *nagariensis* is 1:1, whereas that of *V*. *carteri* f. *kawasakiensis* is 0:1. Thus, *V*. *carteri* f. *nagariensis* can be distinguished from other forms within *V*. *carteri* in producing asexual spheroids with 16 or more gonidia and lacking male spheroids without somatic cells.

*HMG1f* is one of five female-specific genes of *V*. *carteri* f. *nagariensis* (Ferris et al. 2010), and encodes a putative HMG-box DNA binding protein (Umen 2011). In Opisthokonta (Metazoa and Fungi), sex determination involves HMG-box domain proteins (Waters et al. 2007; Idnurm et al. 2008). However, it has been suggested that the female-specific gene *HMG1f* is a possible negative regulator of sexual function in females because its transcription is downregulated in the sexual phase (Umen 2011). The present genomic PCR analyses resolved a possible female strain of *V*. *carteri* f. *nagariensis* (2016-tw-nuk-8-2) that did not exhibit amplification of *HMG1f* (Fig. 4; Additional file 1: Figures S2, S3). This strain exhibited normal asexual and female spheroids that could not be distinguished from those of other female strains (Fig. 5). Thus, *HMG1f* may not contribute to the formation of sexual female spheroids. Further analyses of this strain with sexually active males and other females of *V*. *carteri* f. *nagariensis* are needed to determine the function of *HMG1f.* However, we cannot completely rule out the alternative explanation of polymorphisms in *HMG1f* that precluded amplification. Thus, further analyses are needed for evidencing the complete absence of *HMG1f* in this interesting strain.

## Conclusions

Given that the Japanese and Taiwanese populations of *V*. *carteri* f. *nagariensis* grow in two geographically separated regions (temperate and subtropical, respectively), they can be presumed to be independent lineages derived from a common ancestor. However, the present genomic analyses of 33 Taiwanese strains could not demonstrate the existence of recombination within the > 1 Mbp mating-type locus. This is consistent with previous suggestions that recombination is suppressed within the mating-type locus (Ferris et al. 2010). This may indicate that the > 1 Mbp mating-type locus in *V*. *carteri* f. *nagariensis* is biologically important. Alternatively, such an expanded mating-type locus may result from only passive accumulation of mutations in the duplicated (male and female) genome regions that are divergent enough to inhibit gene conversion (Teshima and Innan 2004). Further studies are needed to resolve this problem, especially in other forms of *V*. *carteri* (Fig. 3).

## Additional file

**Additional file 1: Figure S1.** Alignment of nuclear rDNA ITS-2 sequences used for construction of the phylogenetic tree (Fig. 3). **Figure S2.** Results of genomic PCR of three strains using three pairs of *HMG1f* primers (Table 2 and Additional file 1: Figure S3). Numbers below primer pairs represent expected sizes of the PCR products. Lanes 1, 4 and 7: Eve (UTEX 1885). Lanes 2, 5 and 8: 2016-tw-nuk-8-2. Lanes 3, 6 and 9: 2016-0609-v-1. **Figure S3.** Primer positions of six *HMG1f* primers used in Fig. 3 and Additional file 1: Figure S1.

## Abbreviations

LD: light:dark
nuclear rDNA ITS2: internal transcribed spacer 2 region of nuclear ribosomal DNA
OTU: operational taxonomic unit
PCR: polymerase chain reaction
