# Supplementary material for: Morphology, taxonomy and mating-type loci in natural populations of Volvox carteri in Taiwan
Source: Bot Stud. 2018 Apr 3;59:10. doi: 10.1186/s40529-018-0227-9 (PMC5882469; doi:10.1186/s40529-018-0227-9)
Supplement: Supplementary file 1 — Additional file 1: Figure S1. Alignment of nuclear rDNA ITS-2 sequences used for construction of the phylogenetic tree (Fig. 3). Figure S2. Results of genomic PCR of three strains using three pairs of HMG1f primers (Table 2 and Additional file 1: Figure S3). Numbers below primer pairs represent expected sizes of the PCR products. Lanes 1, 4 and 7: Eve (UTEX 1885). Lanes 2, 5 and 8: 2016-tw-nuk-8-2. Lanes 3, 6 and 9: 2016-0609-v-1. Figure S3. Primer positions of six HMG1f primers used in Fig. 3 and Additional file 1: Figure S1. [file 40529_2018_227_MOESM1_ESM.docx]

**Additional file**

**Morphology, taxonomy and mating-type loci in natural populations of *Volvox carteri* in Taiwan**

**Figures S1-S3.**

**
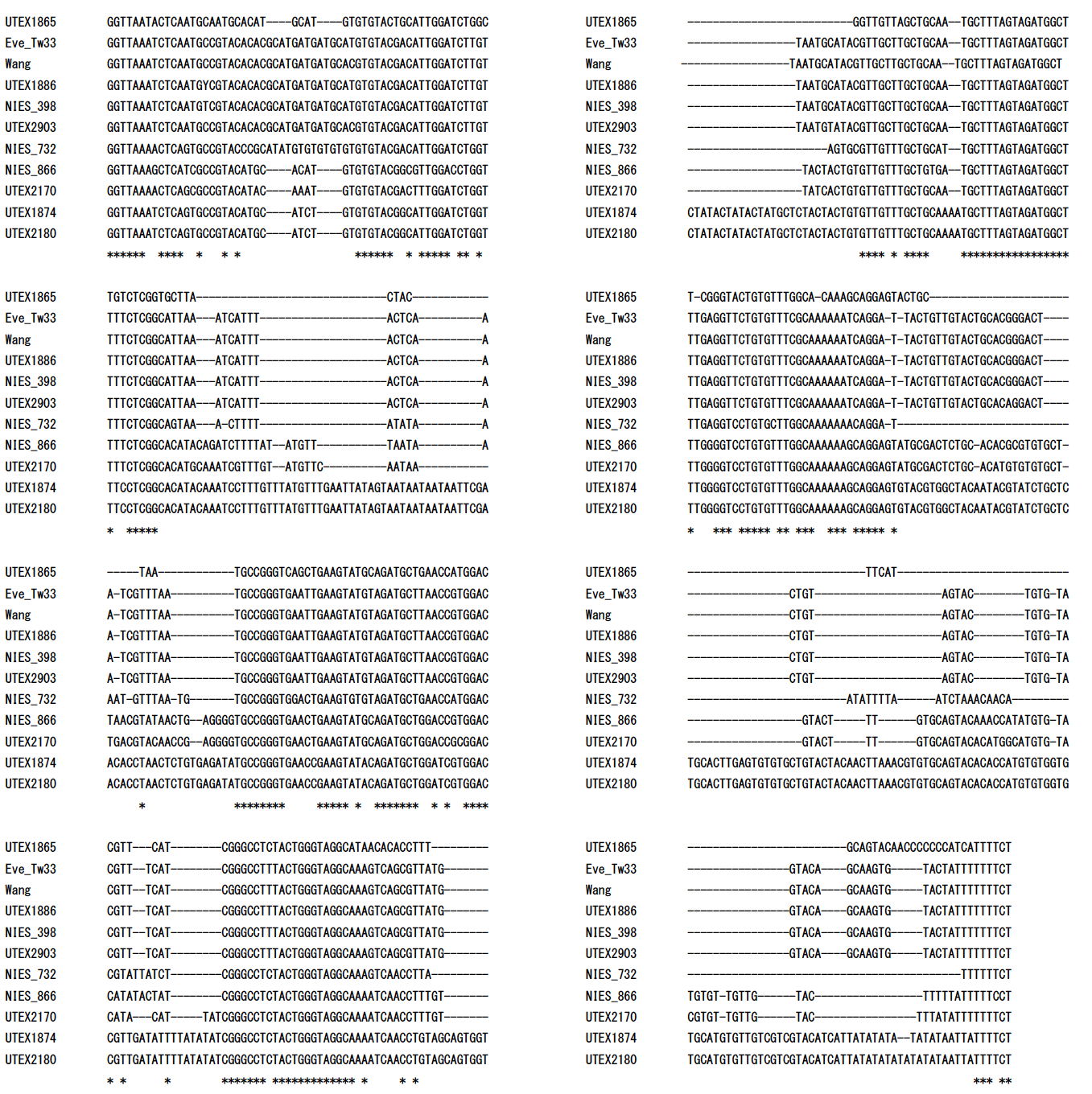
**

Figure S1. Alignment of nuclear rDNA ITS-2 sequences used for construction of the phylogenetic tree (Fig. 3).


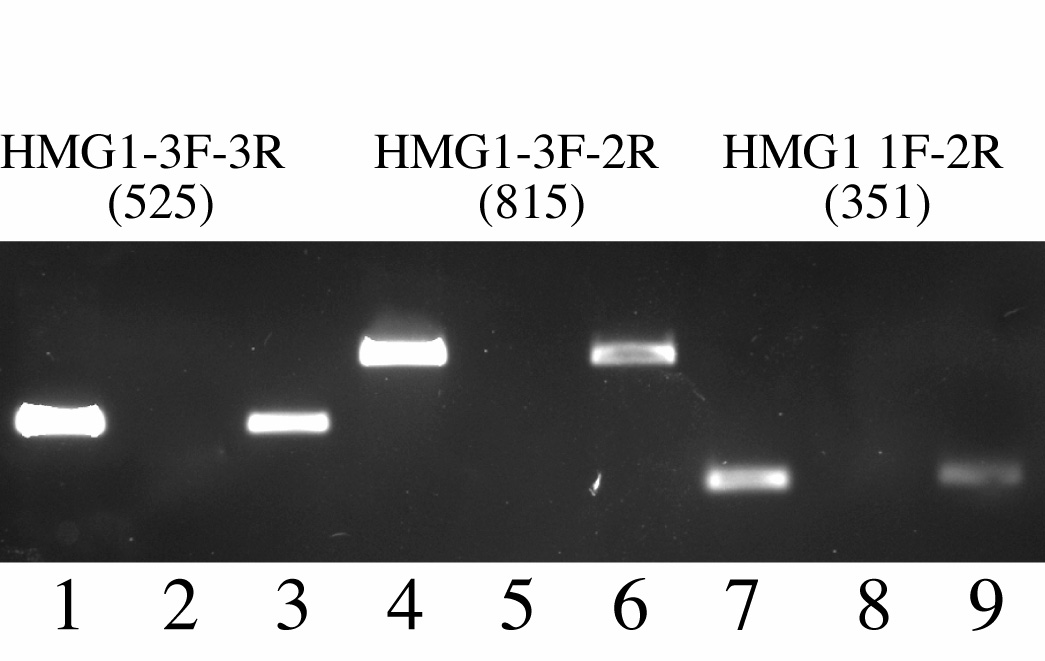


Figure S2. Results of genomic PCR of three strains using three pairs of *HMG1f* primers (Table 2 and Fig. S3). Numbers below primer pairs represent expected sizes of the PCR products. Lanes 1, 4 and 7: Eve (UTEX 1885). Lanes 2, 5 and 8: 2016-tw-nuk-8-2. Lanes 3, 6 and 9: 2016-0609-v-1.


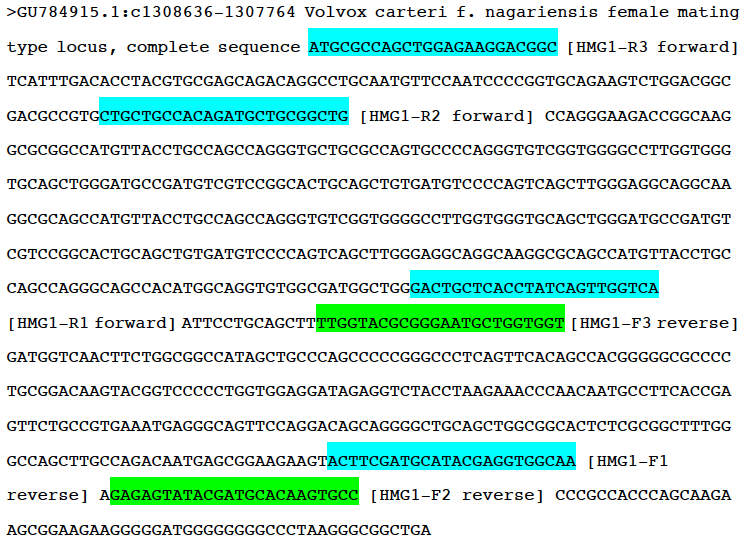


Figure S3. Primer positions of six *HMG1f* primers used in Figure 3 and Figure S1.
